# Cytokine release syndrome after radiation therapy: case report and review of the literature

**DOI:** 10.1186/s40425-017-0311-9

**Published:** 2018-01-03

**Authors:** Christopher A. Barker, Samuel K. Kim, Sadna Budhu, Konstantina Matsoukas, Anthony F. Daniyan, Sandra P. D’Angelo

**Affiliations:** 1 Department of Radiation Oncology, Memorial Sloan Kettering Cancer Center, 1275 York Avenue, New York, NY 10065 USA; 2 Immunology, Memorial Sloan Kettering Cancer Center, New York, USA; 3 Information Systems and Library, Memorial Sloan Kettering Cancer Center, New York, USA; 4 Department of Medicine, Memorial Sloan Kettering Cancer Center, New York, USA

**Keywords:** Cytokine release syndrome, Radiation therapy, Radiotherapy, Systemic inflammatory response syndrome, Cytokine, Tumor necrosis factor, Merkel cell carcinoma, Chronic lymphocytic leukemia, Immunotherapy, Programmed death 1 (PD1)

## Abstract

**Background:**

Cytokine release syndrome (CRS) has been reported after immunologic manipulations, most often through therapeutic monoclonal antibodies. To our knowledge, CRS after radiation therapy (RT) for cancer has not been reported before. The development of unusual clinical signs and symptoms after RT led us to investigate the possibility of CRS after RT and review the medical literature on this topic.

**Case presentation:**

A 65 year-old man with untreated chronic lymphocytic leukemia and recurrent, metastatic Merkel cell carcinoma undergoing anti-programmed death 1 (PD1) immunotherapy was referred for palliative RT to sites of progressing metastases. Within hours of each weekly dose of RT, he experienced fever, tachycardia, hypotension, rash, dyspnea, and rigors. Based on clinical suspicion for CRS, blood cytokine measurements were performed 1 h after the second and third dose of RT and demonstrated tumor necrosis factor alpha (TNF-α) and interleukin-6 (IL-6) levels approximately ten-fold higher than normal. These were near normal immediately prior to the third dose of RT, and resolved to normal levels 3 weeks after RT. He experienced rapid regression of irradiated tumors, with development of new sites of metastases soon thereafter. A literature review revealed no clinical cases of CRS after RT for cancer.

**Conclusions:**

RT during anti-PD1 immunotherapy in a patient with underlying immune dysfunction appeared to be the putative mediator of an immune process which yielded significant increases in pro-inflammatory cytokines, and produced the clinical symptoms meeting the definition of grade 3 CRS. This case demonstrates the capability of RT to elicit immune-related adverse events.

## Background

Cytokine release syndrome (CRS) is defined by the Common Terminology Criteria for Adverse Events (CTCAE, version 4.03) as “a disorder characterized by nausea, headache, tachycardia, hypotension, rash, and dyspnea, and is caused by the release of cytokines by cells” [1]. More specific criteria for CRS are ill-defined, but the condition most likely represents a variant of the Systemic Inflammatory Response Syndrome (SIRS), which is not part of the CTCAE, but was defined by the American College of Chest Physicians and Critical Care Medicine Consensus Conference (ACCP/CCM CC) as the presence of more than one of the following clinical findings: body temperature > 38 °C or <36 °C; heart rate > 90 min^−1^; hyperventilation evidenced by respiratory rate > 20 min^−1^ or PaCO_2_ of <32 mmHg; and a white blood cell count of >12,000 cells mcL^−1^ or <4000 cells mcL^−1^ [2]. Clinical manifestations and laboratory studies (cytokine measurements) can be used to confirm the diagnosis of CRS. Management is often supportive, but patients at risk may benefit from prophylactic antihistamines and corticosteroids. Although a specific mechanistic understanding of CRS and SIRS is unclear, these entities have most often been attributed to immunologic manipulation (in the form of monoclonal antibodies) or insult (in the form of infection), respectively.

To our knowledge, CRS after radiation therapy (RT) has not been described before. Nevertheless, on the basis that RT can modulate the immune system, CRS after RT seems plausible. We recently cared for a patient who developed CRS after RT. For this reason, we report his case in detail and reviewed available literature on CRS and SIRS after RT.

## Case presentation

A 65 year old man presented elsewhere with a 3 cm subcutaneous mass in the right buttock. His past medical history was notable for untreated chronic lymphocytic leukemia (CLL), diagnosed 7 years prior to presentation. He also had coronary artery disease, hypertension, hypothyroidism, and dyslipidemia, all well controlled on medications. Imaging suggested a malignant tumor, and subsequent positron emission tomography (PET) demonstrated 18-fluorodeoxyglucose (FDG) uptake in the right buttock mass, as well as the right inguinofemoral and external iliac lymphatics. Biopsy of the right buttock mass and a right inguinal lymph node demonstrated Merkel cell carcinoma (MCC). Immunohistochemical staining with CM2B4 indicated Merkel cell polyoma virus association with the tumor. Flow cytometric analysis of the lymph node demonstrated a clonal B cell population, consistent with the patient’s known CLL. After diagnosis, his MCC was staged cT2N2M1 and unresectable. He was treated elsewhere with 6 cycles of carboplatin and etoposide chemotherapy. Four weeks after chemotherapy, he was noted to have progression in the in-transit lymphatic metastases, which were biopsied and again revealed MCC.

**Fig. 1:**
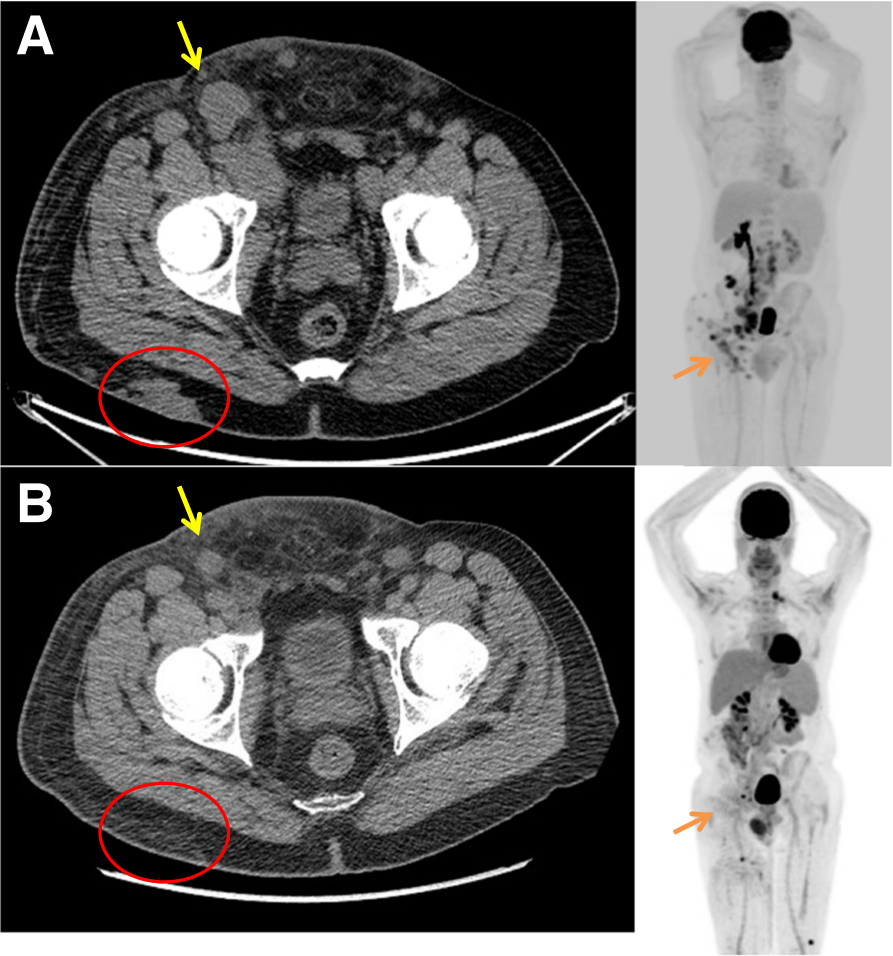
Computed tomography at the level of the pelvis and maximum intensity profile of 18-fluorodeoxyglucose positron emission tomography demonstrates the primary Merkel cell carcinoma tumor of the right buttock (red circle), as well as in-transit metastases and lymphatic metastases (yellow arrow) before (**a**) and after (**b**) radiotherapy. A partial response was noted in the irradiated tumors, with progression of metastases elsewhere

He subsequently presented to our center for evaluation and treatment recommendations. Anti-programmed death 1 (PD1) immunotherapy was recommended, and he received 3 doses of this over 9 weeks at an outside facility without any treatment-related adverse events, including CRS. His in-transit metastases progressed during immunotherapy, and for this reason, he was referred to discuss RT. Physical examination revealed extensive in-transit metastases extending from the right buttock around the upper leg to the anterior leg and groin area, with associated malignant lymphadenopathy and right lower extremity lymphedema. PET/CT corroborated the clinical findings, and also demonstrated metastatic lymphadenopathy involving the right inguinofemoral, external iliac, common iliac and para-aortic lymphatics (see Fig. 1a). Palliative RT was recommended to all sites of grossly evident disease to a total dose of 24 Gy in 3 fractions given once weekly. This radiotherapy regimen has been proven to be safe and effective in a prospective phase II trial [3], and in our experience leads to response in a high proportion of patients with Merkel cell carcinoma [4]. Anti-PD1 therapy was continued during RT.

**Fig. 2:**
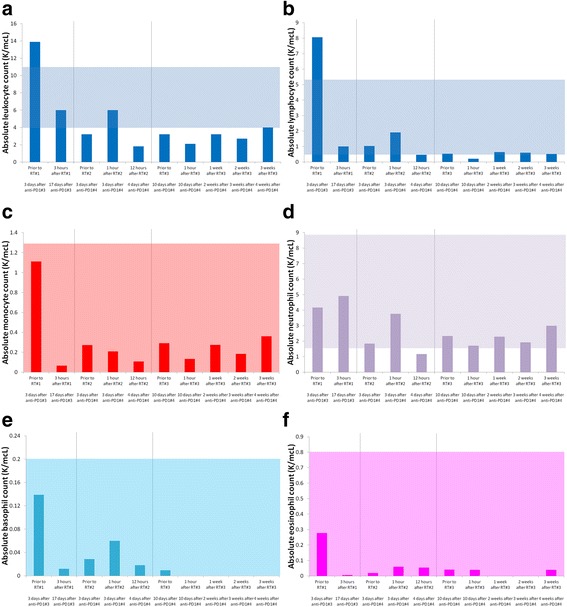
Absolute number of leukocytes (**a**), lymphocytes (**b**), monocytes (**c**), neutrophils (**d**), basophils (**e**), and eosinophils (**f**) before, during, and after radiotherapy and anti-PD1 immunotherapy demonstrate changes in cell counts after each fraction of radiotherapy. Shaded box represents normal range for each cell type

Approximately 1-2 h after his first dose of RT, he developed nausea, vomiting, rigors and dizziness. He presented to another institution and was found to be febrile (temperature 39.1 °C) and tachycardic (heart rate 116 min^−1^). Leukocyte differential is presented in Fig. 2, and demonstrated a dramatic decrease in circulating lymphocytes (Fig. 2b). Blood cultures were obtained and demonstrated no evidence of infection. No signs of organ failure or tumor lysis were detected. He was admitted to another institution for observation and supportive care and discharged <24 h after admission without a specific diagnosis to explain his condition.

**Fig. 3:**
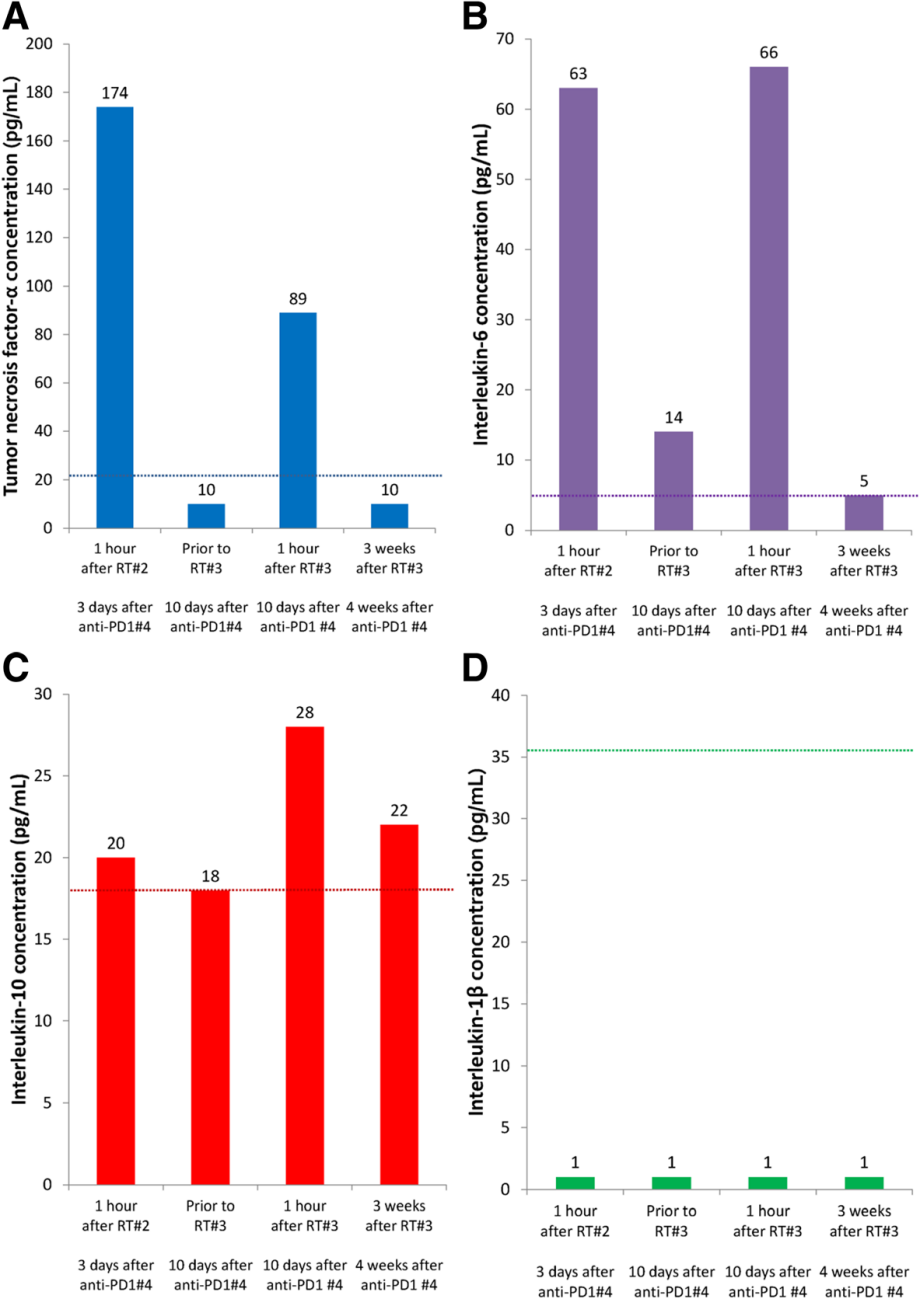
Tumor necrosis factor-α (**a**), interleukin-6 (**b**), interleukin-10 (**c**), and interleukin-1β (**d**) before and after radiotherapy demonstrate increases in cytokines immediately after radiotherapy while the patient was experiencing fever, tachycardia, dyspnea and rigors. Relatively normal levels of cytokines were noted immediately before radiotherapy and 3 weeks after. Dashed lines represent the upper limit of normal for each cytokine

During the 1 week interval between RT treatments, he noted low grade fever, fatigue and a faint maculopapular rash on the trunk. Additional evaluations for infection were unremarkable. He received another dose of anti-PD1 therapy, and this precipitated a low grade fever within hours of infusion. On presentation for his second fraction of RT, it was noted that the in-transit metastases were smaller in size. Given the limited effective alternative treatments available for his MCC, he elected to proceed with the second fraction of RT. Prophylactic antiemetic (ondansetron) and antipyretic (acetaminophen) medications were administered 1 h prior to RT. After RT he was closely observed. One hour after RT he developed violent rigors. He became tachycardic, dyspneic, and hypotensive. CRS was suspected, and laboratory studies (cytokine levels) were requested (Fig. 3). He was admitted to our hospital for <24 h of observation and supportive care. He was again found to have no evidence of infection, organ failure or tumor lysis.

One week later, he presented for his third fraction of RT, and the potential risks, benefits and alternatives to continuing RT were again discussed. He was offered prophylactic immunosuppressants (corticosteroids) as a strategy to prevent CRS. Based on concerns that corticosteroids would abrogate his response to RT, he declined this intervention. Prior to RT, laboratory studies were performed (see Figs. 2 and 3). Approximately 1 h after RT, he again developed rigors, tachycardia, dyspnea, and hypotension. Laboratory tests were repeated, and he was monitored, given supportive care and discharged 6 h later in good condition.

Three weeks after completing RT and receiving no further immunotherapy, he presented to report 2 new skin tumors on the contralateral leg. He had grade 2 radiation dermatitis, grade 1 fatigue, and resolution of his low grade fevers. He reported no other adverse events, including cystitis, enteritis, nausea, vomiting, or diarrhea. PET/CT revealed partial response of the irradiated MCC, with small volume metastatic progression at several new sites (Fig. 1b). Laboratory evaluations were repeated (Figs. 2 and 3). Peripheral blood flow cytometry revealed a clonal B cell population consistent with CLL which represented 4.8% of the peripheral WBCs. The absolute number of CLL cells did not meet diagnostic criteria for the presence of CLL.

## Discussion

As part of the ACCP/CCM consensus definitions statement, it was noted that SIRS “is seen in association with a large number of clinical conditions. Besides the infectious insults that may produce systemic inflammatory response syndrome, noninfectious pathologic causes may include pancreatitis, ischemia, multitrauma and tissue injury, hemorrhagic shock, immune-mediated organ injury, and the exogenous administration of such putative mediators of the inflammatory process as tumor necrosis factor or other cytokines” [2]. In this patient’s case, RT was the putative mediator of the inflammatory process, which elicited increases in tumor necrosis factor-α (TNF-α) and other cytokines, and produced the clinical symptoms and signs observed, which in aggregate met the CTCAE definition of grade 3 CRS as well as SIRS.

It is noteworthy that each fraction of RT precipitated the clinical symptoms and signs of CRS. An assessment of cytokines was performed after the second fraction of RT when CRS was clinically suspected. Moreover, cytokine levels were assessed immediately before and after the third fraction of RT and were found to increase to abnormal levels approximately 1 h after RT, in concert with the development of clinical symptoms. Notably, prophylactic antiemetics (ondansetron) and antipyretics (acetaminophen) were able to prevent the nausea, vomiting and fever observed after the first fraction of RT; however, rigors, tachycardia and dyspnea associated with CRS were not mitigated. In monoclonal antibody therapy, prophylactic corticosteroids and antihistamines are administered to patients at high risk of CRS [5]. Our patient declined this prophylactic intervention. An antibody against TNF-α, such as infliximab, might have mitigated the CRS experienced by this patient.

In our comprehensive review of the published literature, we found no reports of CRS or SIRS after RT for cancer. In a prior phase II trial of the described radiotherapy regimen, CRS was not reported, and grade 3 adverse events occurred in 2 of 18 patients (neuritis, gastrointestinal hemorrhage) [6]. However, prospective studies of patients undergoing total body irradiation prior to bone marrow transplantation have reported some of the clinical manifestations of CRS and SIRS, but neither of these studies formally documented abnormal cytokine levels [7, 8]. A case series of patients with lung cancer reported cytokine levels before and after a protracted course of several weeks of RT, but found no significant changes in cytokine levels; moreover, symptom assessments of CRS or SIRS were not included in that analysis [9]. CRS has been reported after anti-PD1 immunotherapy in case reports [10–12], but does not seem to be common. In our patient anti-PD1 immunotherapy was not temporally associated with CRS.

The mechanism through which RT could cause CRS is unclear. RT likely produced tumor or tissue injury, releasing molecules that express damage-associated molecular patterns causing activated macrophages to release the proinflammatory cytokines, which cause endothelial expression of adhesion molecules and leukocyte extravasation from the periphery at the site of RT. There was no detectable increase in interleukin-1β, which is typically elaborated by activated macrophages in conjunction with TNF-α and interleukin-6. Moreover, the increase in interleukin-10 in conjunction with interleukin-6 suggests these cytokines were functioning in an anti-inflammatory capacity [13]. This patient experienced a precipitous decrease in WBCs and lymphocytes after the first dose of RT (Fig. 2a and b), and the flow cytometric analysis of peripheral lymphocytes 3 weeks after RT revealed minimal residual CLL. However, smaller changes in the WBC or lymphocyte count were observed after fractions 2 and 3 of RT, when CRS was clearly documented (Fig. 2a and b). Moreover, there was no laboratory evidence of tumor lysis syndrome (hyperkalemia, uric acidemia, etc) after fractions 2 or 3 of RT (data not shown). Therefore, it is unlikely that lymphocyte lysis was the primary cause for CRS, as we initially suspected.

A prior study demonstrated that CRS after anti-CD20 monoclonal antibody therapy was most common and severe among patients with CLL that had lymphocyte counts >50 × 10^9^ /L [14]. CRS in these patients was also associated with increases in TNF-α and interleukin-6, with a peak 90 min after infusion. A significant decrease in lymphocyte and platelet counts, and a significant increase in lactate dehydrogenase, liver enzymes and coagulation factors were also noted. However, subsequent doses of antibody therapy were not associated with adverse events. Our case is inconsistent with these results, as our patient’s lymphocyte counts were 13 × 10^9^ /L, yet he developed grade 3 CRS nonetheless. Therefore, pretreatment lymphocyte count may not be a reliable method to predict CRS among patients undergoing RT. In CLL, lymphocyte counts >50 × 10^9^ /L represent a significant volume of malignant cells; in our patient’s case, the large volume of MCC may have been the risk factor for CRS. We suspect that when large volumes of malignant cells are killed by effective cancer therapies, the risk of CRS is greatest.

The underlying immune dysfunction caused by CLL, and the ongoing immunotherapy with anti-PD1 therapy may have exacerbated what may typically be a subtle immunologically mediated response to cancer RT, although this is speculation requiring further investigation. Previous work has suggested that proinflammatory cytokine release by T cells is enhanced with PD-1 axis blockade [15]. Moreover, patients with untreated CLL and MCC are known to have an expanded myeloid derived suppressor cell (MDSC) compartment [16] and MDSC compartment at the tumor microenvironment interface [17], respectively. RT may have rapidly depleted MDSCs and allowed for unchecked elaboration of pro-inflammatory cytokines [18]. The pervasive innervation of the anatomic region of RT in this case (abdomen and pelvis) by the vagus nerve may also be a contributor to the autonomic physiologic response observed during CRS [19]. Finally, it is possible the advanced age of the patient (65 years) may have contributed to the observation of an atypical immune response to cancer therapy [20]. Because CRS after RT has never been reported before, it is possible that the unusual combination of circumstances (untreated CLL, antiPD1 immunotherapy, large volume MCC, advanced patient age) may have contributed to the development of CRS after RT.

The observation of rapid response of irradiated metastases and the development of new metastases after CRS is also of interest. Several studies have suggested the prometastatic and proinvasive effects of RT, and this may be mediated in part by PI3K/Akt-signaling pathway and NF-kB activation and induction of inflammatory cytokines such as TNF-α and interleukin-6 [21–24]. Furthermore, studies have also noted differences in disease prognosis based on the expression levels of various cytokines in numerous cancers, where higher levels of interleukin-6 and interleukin-10 were associated with poorer outcomes [25–27]. Further study of the acute cytokine release after RT with or without immunotherapy as well as the associated molecular pathways may help clarify the mechanisms of metastasis response and resistance to therapy.

While CRS is most often attributed to immunomodulatory monoclonal antibodies, it is possible that other immunomodulatory cancer therapies (such as RT) could produce this effect [28]. While some regard RT as an immunosuppressant, we interpret the observations of this patient’s case as evidence that RT is best described as an immune-modulator, not with exclusive suppressive functions. Consistent with this, the immunomodulatory effects of RT have been associated with differences in the radiation dose delivered during treatment [29]. In an era of increasing interest in combining RT and immunotherapy for cancer, careful monitoring for immune-related adverse events not typically observed with RT or immunotherapy alone will probably be of great importance [30]. Clinicians and investigators alike are encouraged to consider how these agents may interact in both positive and negative ways to further our understanding of this promising therapeutic combination.

## Conclusions

To our knowledge, we are the first to report CRS after the receipt of RT. RT in a patient with underlying immune dysfunction who was concurrently receiving anti-PD1 immunotherapy appeared to have been the likely mediator of this immune process. This immune process, in turn, generated significant increases in pro-inflammatory cytokines and produced the clinical symptoms meeting the definition of grade 3 CRS. Thus, this case demonstrates the capability of RT to elicit immune-related adverse events and allows us to gain greater insight into the therapeutic combination of RT and immunotherapy.

## References

[1] National Cancer Institute Common Terminology Criteria for Adverse Events v4.0 NCI, N., DHHS. May 29, 2009 NIH publication # 09–7473.

[2] Bone RC, et al. Definitions for sepsis and organ failure and guidelines for the use of innovative therapies in sepsis. The ACCP/SCCM consensus conference committee. American College of Chest Physicians/Society of Critical Care Medicine. Chest. 1992;101(6):1644–55. 10.1378/chest.101.6.1644 1303622

[3] Salama JK, Chmura SJ, Mehta N, Yenice KM, Stadler WM, Vokes EE, Haraf DJ, Hellman S, Weichselbaum RR. An initial report of a radiation dose-escalation trial in patients with one to five sites of metastatic disease. Clin Cancer Res. 2008;14(16):5255–9. 10.1158/1078-0432.CCR-08-0358 18698045

[4] Cimbak N, Barker CA (2016). Short-course radiation therapy for Merkel cell carcinoma: relative effectiveness in a "radiosensitive" tumor. International Journal of Radiation Oncology Biology Physics.

[5] Breslin S (2007). Cytokine-release syndrome: overview and nursing implications. Clin J Oncol Nurs.

[6] Salama JK (2012). Stereotactic body radiotherapy for multisite extracranial oligometastases final report of a dose escalation trial in patients with 1 to 5 sites of metastatic disease. Cancer.

[7] Buchali A (2000). Immediate toxicity during fractionated total body irradiation as conditioning for bone marrow transplantation. Radiother Oncol.

[8] Chaillet MP (1993). Prospective study of the clinical symptoms of therapeutic whole body irradiation. Health Phys.

[9] Trovo M (2016). Stereotactic body radiation therapy and intensity modulated radiation therapy induce different plasmatic cytokine changes in non-small cell lung cancer patients: a pilot study. Clin Transl Oncol.

[10] Foran AE (2017). Nivolumab in the treatment of refractory pediatric Hodgkin lymphoma. J Pediatr Hematol Oncol.

[11] Rotz SJ (2017). Severe cytokine release syndrome in a patient receiving PD-1-directed therapy.

[12] Rassy EE (2017). Diffuse edema suggestive of cytokine release syndrome in a metastatic lung carcinoma patient treated with pembrolizumab. Immunotherapy.

[13] Schaue D, Kachikwu EL, McBride WH (2012). Cytokines in radiobiological responses: a review. Radiat Res.

[14] Winkler U (1999). Cytokine-release syndrome in patients with B-cell chronic lymphocytic leukemia and high lymphocyte counts after treatment with an anti-CD20 monoclonal antibody (rituximab, IDEC-C2B8). Blood.

[15] Dirks J (2013). Blockade of programmed death receptor-1 signaling restores expression of mostly proinflammatory cytokines in anergic cytomegalovirus-specific T cells. Transpl Infect Dis.

[16] Jitschin R (2014). CLL-cells induce IDOhi CD14+HLA-DRlo myeloid-derived suppressor cells that inhibit T-cell responses and promote TRegs. Blood.

[17] Mitteldorf C (2017). PD-1 and PD-L1 in neoplastic cells and the tumor microenvironment of Merkel cell carcinoma. J Cutan Pathol.

[18] Postow MA (2012). Immunologic correlates of the abscopal effect in a patient with melanoma. N Engl J Med.

[19] Tracey KJ (2007). Physiology and immunology of the cholinergic antiinflammatory pathway. J Clin Invest.

[20] Saavedra D, Garcia B, Lage A (2017). T cell subpopulations in healthy elderly and lung cancer patients: insights from Cuban studies. Front Immunol.

[21] Su WH (2012). Radiation-induced increase in cell migration and metastatic potential of cervical cancer cells operates via the K-Ras pathway. Am J Pathol.

[22] Qian LW (2002). Radiation-induced increase in invasive potential of human pancreatic cancer cells and its blockade by a matrix metalloproteinase inhibitor, CGS27023. Clin Cancer Res.

[23] Cheng JC (2006). Radiation-enhanced hepatocellular carcinoma cell invasion with MMP-9 expression through PI3K/Akt/NF-kappaB signal transduction pathway. Oncogene.

[24] Wild-Bode C (2001). Sublethal irradiation promotes migration and invasiveness of glioma cells: implications for radiotherapy of human glioblastoma. Cancer Res.

[25] Voorzanger N (1996). Interleukin (IL)-10 and IL-6 are produced in vivo by non-Hodgkin's lymphoma cells and act as cooperative growth factors. Cancer Res.

[26] Suzuki S (2001). IL-6 and IFN-gamma regulation of IL-10 production by human colon carcinoma cells. Int J Oncol.

[27] Bien E (2009). Pre-treatment serum levels of interleukin-10, interleukin-12 and their ratio predict response to therapy and probability of event-free and overall survival in childhood soft tissue sarcomas, Hodgkin's lymphomas and acute lymphoblastic leukemias. Clin Biochem.

[28] Suntharalingam G (2006). Cytokine storm in a phase 1 trial of the anti-CD28 monoclonal antibody TGN1412. N Engl J Med.

[29] Janiak MK (2017). Cancer immunotherapy: how low-level ionizing radiation can play a key role.

[30] Escorcia FE, Postow MA, Barker CA (2017). Radiotherapy and immune checkpoint blockade for melanoma: a promising combinatorial strategy in need of further investigation. Cancer J.

